# Non-response bias in the Norwegian Counties Public Health Survey: Insights from linkage to register data

**DOI:** 10.1177/14034948251360674

**Published:** 2025-08-06

**Authors:** Thomas S. Nilsen, Marit Knapstad, Jens C. Skogen, Leif E. Aarø, Øystein Vedaa

**Affiliations:** 1 Department of Mental Health, Norwegian Institute of Public Health, Oslo, Norway; 2 Department of Health Promotion, Norwegian Institute of Public Health, Bergen, Norway; 3 Centre for Evaluation of Public Health Measures, Norwegian Institute of Public Health, Oslo, Norway; 4 Centre for Alcohol and Drug Research (KORFOR), Stavanger University Hospital, Norway; 5 Department of Psychosocial Science, University of Bergen, Norway

**Keywords:** Non-response bias, public health surveys, health surveys. cross-sectional studies, registry data, disability pensions, sick leave, work assessment allowance, mental health, socioeconomic factors

## Abstract

**Aims:**

The Norwegian Counties Public Health Surveys (NCPHS) aim to capture critical aspects of the population’s health and well-being. However, selective non-response can introduce bias, potentially compromising survey representativeness. This study assesses non-response patterns in NCPHS using registry data on health-related benefits.

**Methods:**

NCPHS comprises cross-sectional web-based surveys assessing various health metrics in Norway’s adult population. This study uses data from NCPHS conducted in Hordaland County (April 10 to May 17 2018). Health data, including sickness benefits, disability pension and work assessment allowance (WAA), were linked from national registries.

**Results:**

From 34,925 invited residents, the response rate was 45.3%, with higher rates among females (50.0%), older individuals (61.0% for ages 60–69 years) and those with higher education (55.9%). Sickness benefit was not associated with participation rates (adjusted risk ratio (RR) 0.96 I, 95% confidence interval (CI): 0.90–1.02). Disability pension, particularly for mental and behavioural disorders, was associated with lower response rates (adjusted RR 0.81, 95% CI: 0.72–0.92). WAA had no overall significant effect, but WWA due to psychological conditions was related to lower response propensity (adjusted RR 0.85, 95% CI: 0.73–0.99).

**Conclusions:**

**These findings suggest that health-related non-response associated with these benefits has a limited impact on overall population estimates but may introduce bias in specific subgroups, depending on the research question. Though the degree selection into a survey creates bias depend on the research question, knowing that there is relatively little selection by factors such as sickness benefit, WAA and disability pension is important as 22–23% of the population aged 18–67 receive one of these benefits at any time.**

## Background

Population health surveys are widely used to monitor health and wellbeing at the local and national levels. In Norway, the Norwegian Counties Public Health Surveys (NCPHS) were developed to support this aim, providing data to inform local policy [1]. However, such surveys rely on voluntary participation, and non-response may lead to biased estimates if participation is systematically related to factors such as health status or social disadvantage [2–4]. This concern is not unique to Norway or to the NCPHS: understanding who does not respond – and why – remains a key challenge in population health research [4–6]. In this study, we use registry data to examine whether certain health-related social benefits are associated with survey participation, offering insights that extend beyond the immediate survey context.

Response rate alone is not a reliable indicator of representativeness [4,7,8] .Previous research using this survey material found minimal differences between early and late responders on key health indicators [9], suggesting that variations in participation propensity do not always compromise representativeness. The key concern in the present paper is whether non-response is systematically related to characteristics that also may affect outcomes of interest. For example, if individuals with poorer health are less likely to participate, estimates of population health may be biased. From our surveys, and other, similar, surveys, it is well established that younger people have lower probability of responding than older people, and that men respond at lower rates than women [1]. Response propensity also follows an education gradient, with higher response rates among those with higher levels of education. Response propensity is also related to other core sociodemographic characteristics such as employment status and income [9–11]. These patterns can in many cases be accommodated using post-stratification weights, as the demographic composition of the target population is known from official statistics. However, non-response may also be linked to other factors that are not observed among non-responders, for example those related to health status – which limits our ability to assess or correct for potential selection bias. Previous studies have shown that health may influence response propensity, with poorer health being associated with a lower likelihood of response [2,3,12–15]. To examine whether this is the case, we need more information about non-responders, especially regarding factors that may both affect response propensity and relate to key health outcomes. In the context of NCPHS, our primary interest is to examine whether response propensity is associated with health-related characteristics that are themselves relevant to the survey’s core aims. By using registry data on health-related benefits receipt, we can explore this potential mechanism of selection bias in a way that is not possible with survey data alone.

## Aims

The aim of this study is to assess patterns of non-response in NCPHS by examining whether participation is associated with indicators of health. Using individual-level linkages to national registry data, we investigate whether receipt of sickness benefits, work assessment allowance (WAA) or disability pension – as proxies for physical and mental health impairments – is related to the likelihood of responding to the survey. In doing so, we assess the extent to which health-related selection mechanisms may affect the representativeness of data from public health surveys.

## Materials and methods

The NCPHS comprises cross-sectional surveys conducted county-wise by the Norwegian Institute of Public Health, assessing health, health behaviours, social inclusion, quality of life, and local context health-related factors in Norway’s adult population [1]. The present study is based on data from this 2018 survey, conducted in Hordaland County. To assess patterns of non-response, we used registry linkage to combine information from the survey with administrative data on benefit receipt and sociodemographic characteristics. The study was approved by the Norwegian Data Protection Authority. No approval from the Regional Committees for Medical and Health Research in Norway was necessary, as this study does not fall under the purview of Norway’s Health Research Act.

A random sample consisting of 38,743 residents from Hordaland County was drawn from the National Population Register for participation in the survey (6.1% of the eligible population). Eligibility required individuals to be aged over 18 years and to have their mobile phone number and email address registered in the Common Contact Register (Norwegian Digital Agency). The sample size aimed for adequate subgroup analyses on a municipal level and city districts in Bergen City (the largest city in Hordaland County). The survey, accessible via PCs, mobile phones and tablets, was distributed via email and SMS, with reminders sent after seven and 15 days. The full sample, including both responders and non-responders, was subsequently linked to national registries using personal identification numbers, enabling individual-level analyses of participation patterns. In this paper, the term ‘survey period’ refers to the data collection window: April 10 to May 17 2018.

Indicators of mental and physical health were derived from national registry data. All Norwegian citizens have a personal identification number that enables linkage across administrative sources. The full invited sample was linked to individual-level data from Statistics Norway (for education and employment) and the Norwegian Labour and Welfare Administration (receipt of sickness benefits, work assessment allowance (WAA) and disability pension) [16,17]. The three health-related benefits reflect different types and durations of reduced work capacity and serve as proxies for physical and mental health status in the analysis.

### Measures

Sickness benefit is available to individuals in the National Insurance Scheme with legal residence in Norway and at least four weeks of prior employment. Sickness benefit is given from the first sickness day and can be extended up to one year. Sickness benefits in the current analyses are medically certified sickness absences. If the worker is not able to return to work within a year, or if not eligible for sickness benefit owing to income requirements, WWA might be granted for up to three years (four years before 2018). WAA is a temporary benefit intended for individuals with prolonged reduced work capacity who are undergoing medical treatment, rehabilitation or work-related follow-up. Disability pension provides long-term income support for individuals with permanent work disability and has an upper age limit at 67 years, when recipients transition to retirement pension. One will normally receive only one of these benefits at any given time. However, WAA and disability pension can be given gradually and combined with work, and it is also permissible to work even when granted 100% WAA and disability pension, within certain limits.

The medico-legal reason for sickness benefits, disability pension and WAA are coded based on the International Statistical Classification of Diseases (ICD) and International Classification of Primary Care (ICPC). WAA was predominantly coded in ICPC-1/2; cases coded in ICD-9/10 were recoded to corresponding ICPC codes according to guidelines from the Norwegian Directorate of health [18]. Sickness benefit was coded in ICPC and disability pension in ICD-10.

For analysis, individuals were coded as sickness benefit recipients (1) if they had at least one day of medically certified sick leave during the survey period, and 0 otherwise. The reference group for the sickness benefit analysis was the working-age population (⩽70 years) who were part of the workforce during the survey period but did not receive sickness benefit. Individuals who were on WAA or disability pension but also had wage income during the survey period were retained in the sickness benefit model and coded as 0 (i.e. eligible but not on sickness benefit).

Long-term sick leave was defined as a duration of eight weeks or more, in accordance with the National Insurance Act [19], which requires a more comprehensive medical assessment at that point. Individuals meeting this threshold during the survey period were classified as long-term sickness benefit recipients.

We distinguished between current disability pension (ongoing receipt at the time of the survey) and previous disability pension (terminated owing to transition to retirement pension at age 67 years). As the medico-legal grounds for disability pension typically persist after retirement, both current and previous disability pension were included in the analysis [20]. Individuals were coded as 1 for current or previous disability pension depending on their status, and 0 otherwise. For disability pension, the reference group comprised non-recipients, stratified by age: those under 67 for comparisons with current disability pension, and those 67 and older for comparisons with previous disability pension (i.e. transitioned to retirement).

WAA was coded as 1 if granted at any time during the survey period, and 0 otherwise. Reference group included individuals under 67 years who were not receiving disability pension. Since WAA and DP are universal benefits not contingent on employment, no work history restrictions were applied to these models.

Approximately two-thirds of benefit recipients had diagnoses related to musculoskeletal and mental health conditions. To ensure sufficient statistical power, diagnostic codes were grouped into three categories: for ICPC-coded sickness benefits and WAA, these included chapter L (musculoskeletal), chapter P (psychological) and all others; for ICD-coded disability pension, these corresponded to chapter XIII (diseases of the musculoskeletal system and connective tissue), chapter V (mental and behavioural disorders) and all other chapters.

Workforce participation was coded as 1 for individuals with registered wage income or formal unemployment during the survey period, and 0 otherwise. This measure was used to define eligibility for sickness benefits, which are tied to recent labour market activity. Education was categorized using the Norwegian Standard Classification of Education into five groups: basic school, upper secondary, tertiary vocational, short higher education (⩽4 years) and long higher education (>4 years). Both variables were included as covariates in the adjusted models [21].

### Statistical analysis

Descriptive statistics of response rates are presented for the sample stratified on age, sex and education. The primary outcome variable was survey participation (yes/no). Given the high overall response rate, we used Poisson regression with robust standard errors to estimate risk ratios (RRs), as this approach provides more accurate effect estimates than odds ratios, which tend to be inflated for common outcomes [22].

Analyses were conducted separately for sickness benefits, WAA and disability pension. For each, participation was modelled as a function of benefit status, with age, sex and education entered as controls, as they are strongly associated with response propensity and the independent variables [23]. Diagnostic subgroups (psychological, musculoskeletal, other) were analysed within each benefit category using ICD or ICPC codes, as described above.

## Results

From a sample of 34,925 invited participants, the overall response rate was 45.3%. Females responded at a higher rate (50.0%) compared with males (41.0 %). Response rates increased with age and education, from 31.4% among those aged 18–29 to 61.0% among those aged 60–69 years, and from 32.0% among those with basic education to 55.9% among those with higher education (>4 years). These descriptive patterns are shown in Table I.

**Table I.** Survey participation by gender, age and education.

Sample	Invited	Responded	Response rate (%)
Sex			
Female	16,923	8462	50.0
Male	18,002	7373	41.0
Age group			
18–29	8378	2630	31.4
30–39	6859	2627	38.3
40–49	6628	3026	45.7
50–59	5762	3189	55.4
60–69	4614	2815	61.0
70–79	2172	1294	59.6
80+	512	254	49.6
Education^a^			
Basic school level	5704	1827	32.0
Upper secondary school level	13,464	5983	44.4
Tertiary vocational education	1347	672	49.9
Higher education, short (⩽4 years)	9400	5068	53.9
Higher education, long (>4 years)	3667	2051	55.9
Total	34,925	15,835	45.3

a The category ‘no registered education’, comprising 29 persons, is left out.

Among the invited participants, 1417 (4.2%) had at least one registered sickness benefit during the survey period, with a response rate of 45.2% (Table II). In adjusted Poisson regression models, receipt of sickness benefit was not significantly associated with survey participation (adjusted RR 0.96, 95% confidence interval (CI): 0.90–1.02). No significant differences were observed by diagnostic category. Long-term sick leave (>8 weeks) was also not associated with lower participation (adjusted RR 0.93, 95% CI: 0.85–1.01).

**Table II.** Sickness benefits by ICPC disease chapter: response rates and risk ratios.

Disease chapter – ICPC	Invited	Responders	Response rate (%)	RR (95% CI)	Adjusted RR (95% CI)^a^
No SB^b^	25,095	11,330	45.2	Ref.	Ref.
L Musculoskeletal	547	245	44.8	0.99 (0.90–1.09)	0.96 (0.87–1.05)
P Psychological	305	141	46.2	1.02 (0.91–1.16)	0.96 (0.85–1.08)
Other^c^	472	222	47.0	1.04 (0.95–1.15)	0.96 (0.88–1.06)
Any SB	1417	641	45.2	1.02 (0.96–1.08)	0.96 (0.90–1.02)
Long term SB (Ref.: no SB )	672	304	45.2	1.00 (0.92–1.09)	0.93 (0.85–1.01)

a Adjusted for age, sex and education.

b Reference group is those eligible for SB, that is, registered as part of the workforce in the period of interest, but not registered with SB during this period.

c Letters A to Y in the ICPC classification, except L and P.

ICPC: International Classification of Primary Care; RR: risk ratio; CI: confidence interval; SB: sickness benefits; Ref.: reference.

A total of 797 individuals received WAA during the survey period, with a response rate of 44.5% (Table III). Overall, WAA receipt was not significantly associated with survey participation after adjustment (RR 1.00, 95% CI: 0.92–1.08). However, stratified analysis by diagnosis revealed a lower likelihood of response among individuals receiving WAA for psychological conditions (ICPC chapter P), with an adjusted RR of 0.85 (95% CI: 0.73–0.99). No significant associations were observed for musculoskeletal (chapter L) or other diagnoses. Among the 1334 participants with current disability pension, the overall response rate was 44.9% (Table IV). In adjusted models, individuals with current disability pension due to mental and behavioural disorders (ICD-10 chapter V) were less likely to participate compared with those aged 18–66 years without disability pension (adjusted RR 0.81, 95% CI: 0.72–0.92). No significant differences in participation were observed among those with musculoskeletal diagnoses (chapter XIII, adjusted RR 0.92, 95% CI: 0.84–1.02) or other conditions (adjusted RR 0.93, 95% CI: 0.85–1.02). The aggregate adjusted RR for all individuals with current disability pension was 0.89 (95% CI: 0.84–0.95). Among those aged 67 years and older, a subgroup of 338 individuals had previously received disability pension before transitioning to retirement. This group also showed lower participation, particularly among those with a history of disability pension for mental and behavioural disorders (adjusted RR 0.73, 95% CI: 0.56–0.94). The overall adjusted RR for previous disability pension in this age group was 0.84 (95% CI: 0.75–0.94).

**Table III.** Work assessment allowance by ICPC disease chapter: response rates and risk ratios.

Disease chapter – ICPC	Invited	Responders	Response rate (%)	RR (95% CI)	Adjusted RR (95% CI)^a^
No WAA^b^	28,151	12,258	43.5	Ref.	Ref.
L Musculoskeletal	244	115	47.1	1.08 (0.95–1.23)	1.04 (0.92–1.18)
P Psychological	322	110	34.2	0.79 (0.68–0.92)**	0.85 (0.73–0.99)*
Other^c^	231	120	52.0	1.21 (1.07–1.36)**	1.13 (1.00–1.27)
Any WAA (Ref.: no WAA)	797	345	44.5	1.00 (0.93–1.08)	1.00 (0.92–1.08)

a Adjusted for age, sex and education.

b All below 67 years and with no disability pension.

c All ICPC chapters except P and L.

* *p* < 0.05.

** *p* < 0.01.

ICPC: International Classification of Primary Care; RR: risk ratio; CI: confidence interval; WAA: work assessment allowance; Ref.: reference.

**Table IV.** Disability pension by ICD-10 chapter: response rates and adjusted risk ratios.

ICD-10 chapter	Invited	Responders	Response rate (%)	RR (95% CI)	Adjusted RR (95% CI)^a^
**Current DP**					
No DP^b^	29,670	12,926	43.57	Ref.	Ref.
V – mental and behavioural disorders	463	170	36.72	0.84 (0.75–0.95)*	0.81 (0.72–0.92)*
XIII– diseases of the musculoskeletal system and connective tissue	418	210	50.24	1.15 (1.05–1.27)*	0.92 (0.84–1.02)
Other^c^	463	223	48.16	1.11 (1.00–1.22)**	0.93 (0.85–1.02)
Any current DP	1334	603	44.87	1.03 (0.97–1.09)	0.89 (0.84–0.95)*
**Previous DP**					
No DP^d^	3573	2135	59.75	Ref.	Ref.
V – mental and behavioural disorders	63	29	46.03	0.77 (0.59–1.01)	0.73 (0.56–0.94)**
XIII – diseases of the musculoskeletal system and connective tissue	148	73	49.03	0.83 (0.70–0.97)*	0.86 (0.73–1.02)
Other^c^	127	69	54.33	0.91 (0.77–1.07)	0.87 (0.75–1.02)
Any previous DP	338	171	50.59	0.85 (0.76–0.94)*	0.84 (0.75–0.94)*

a Adjusted for age, sex and education.

b All below 67 years and with no DP.

c All ICD chapters except V and XIII.

d >66 years and no previous DP.

* *p* <0.01.

** *p* <0.05.

ICD: International Statistical Classification of Diseases; RR: risk ratio; CI: confidence interval; DP: disability pension; Ref.: reference.

## Discussion

The purpose of this study was to assess non-response patterns in NCPHS by comparing health indicators of responders and non-responders using registry data on benefits and allowances. Our findings revealed no statistically significant difference in participation rates between invited individuals receiving sickness benefits and those without, suggesting that receiving sickness benefits does not predict participation in web surveys in the working age population. Similarly, survey response was not predicted by sickness benefits diagnostic categories or the duration of sickness benefits. Individuals receiving disability pension – both current recipients and those who had transitioned to retirement – were less likely to participate in the survey compared with others in their respective age groups. This was particularly evident among those with mental and behavioural disorders (ICD-10 Chapter V). This disparity may reflect the more severe, chronic and functionally impairing conditions required for disability pension eligibility, in contrast to the more transient or acute illnesses typically covered under sickness benefits. Moreover, disability pension recipients tend to have lower levels of education and income, both of which are independently associated with lower response propensity [24]. Together, these factors are likely to contribute to a pattern of health-related selection bias in survey participation. Discrepancies between crude response rates and adjusted regression estimates – such as for disability pension – reflect the influence of covariates such as age, sex and education. Adjusted models isolate the association between benefit status and participation after accounting for these confounding factors.

WAA did not show an overall association with response rates, indicating that participation among recipients of this transitional benefit is broadly similar to the general population. However, individuals receiving WAA for psychological diagnoses had a slightly lower likelihood of responding. Because of statistical power considerations, all psychological diagnoses were analysed as an aggregated category, and further disaggregation was not feasible. While this limits diagnostic specificity, it supports a broader interpretation that persistent psychological conditions may be associated with lower survey participation. Given the population-level focus of the NCPHS, these findings highlight a structural pattern rather than case-level diagnostic effects. The main findings are in line with two studies having analysed sickness benefits and disability pension in relation to non-response in similar, general population-based settings [14,15]. In one of these, the effect of sickness benefits on response propensity was deemed significant in statistical terms, but insignificant in practical terms and impact [15]. The other study found a stronger effect for disability pension (current and future), especially for mental disorders, with a relative risk of non-participation of 2.98 (95% CI 2.87–3.10) [14]. It should be noted that this analysis was done on a sample from the same county as the present study, years 1997–1999, and included physical examinations. The requirement to attend for a physical examination might have been a barrier for those with poorer health and could partly explain the high relative risk of non-response in that particular study.

Patterns of non-response are often described using the framework of Missing Completely at Random (MCAR), Missing at Random (MAR) and Missing Not at Random (MNAR) [4,25]. In practice, MCAR is rarely plausible. In our case, if participation is influenced by underlying health status – such as psychological conditions linked to WAA or disability pension – this might represent a MNAR mechanism, where the probability of missingness is related to the outcome of interest (e.g. psychological distress). Traditional adjustment methods such as weighting may not fully correct for this. However, access to registry data on benefit status allows us to observe key predictors of non-response, moving the analysis closer to a MAR scenario, where such bias can be more effectively addressed. While our findings suggest limited overall impact on public health estimates, the partial under-representation of individuals with disabling mental health conditions should be acknowledged, particularly since 22–23% of the working-age population receives one of these benefits [23].

### Limitations

The main strength of the present analysis is the use of individual-level registry data from both participants and non-participants. One concern is that web-based surveys may exclude non-digital citizens. Invitations to the NCPHS are sent via SMS and email using the Common Contact Register (CCR), which requires prior use of certain digital public services. Coverage in the CCR is not complete, especially among older adults [26]. Consequently, web-based surveys may under-represent frailer segments of the elderly population and overestimate overall health in this group [27].

As noted above, sickness benefits in this study refers only to medically certified sickness absences. Around 17% of all sick leave is self-certified and not associated with a recorded diagnosis [28], and many individuals with health complaints do not take sick leave at all [29]. These gaps might conceal unmeasured sources of selection. In addition, our analysis does not account for health problems that do not result in sickness benefits, WAA or disability pension. For example, we lack information on alcohol use, which is known to influence response rates and can bias estimates of consumption at the population level [30]. If individuals with heavier alcohol use are less likely to respond, this may lead to underestimation of mental health conditions such as anxiety and depression that are often associated with alcohol use but not consistently recorded in benefit registries [31]. As discussed earlier, mechanisms of missingness that are not fully explained by observed variables (i.e. MNAR or partially observed MAR) are likely to remain. Analyses such as the present one help narrow the scope of unobserved bias, but do not eliminate it.

## Conclusion

In this study of 34,925 individuals, the response rate was 45.3%. Females, older adults, and those with higher education were more likely to respond. Receiving sickness benefits or WAA overall was not associated with participation, but lower response was observed among those receiving disability pension, and especially among WAA or disability pension recipients with mental and behavioural disorders. These findings suggest that health-related non-response associated with these benefits has a limited impact on overall population estimates but may introduce bias in specific subgroups, depending on the research question.
